# Biomarkers for Kawasaki Disease: Clinical Utility and the Challenges Ahead

**DOI:** 10.3389/fped.2019.00242

**Published:** 2019-06-18

**Authors:** Himanshi Chaudhary, Johnson Nameirakpam, Rajni Kumrah, Vignesh Pandiarajan, Deepti Suri, Amit Rawat, Surjit Singh

**Affiliations:** Post Graduate Institute of Medical Education and Research, Chandigarh, India

**Keywords:** immunology, vasculitis, Kawasaki, biomarkers, childhood vasculitis

## Abstract

Kawasaki disease (KD) has replaced acute rheumatic fever as the most common cause of acquired heart disease in children in the developed world and is increasingly being recognized from several developing countries. It is a systemic vasculitis with a predilection for coronary arteries. The diagnosis is based on a constellation of clinical findings that appear in a temporal sequence. Quite understandably, this can become a problem in situations wherein the clinical features are not typical. In such situations, it can be very difficult, if not impossible, to arrive at a diagnosis. Several biomarkers have been recognized in children with acute KD but none of these has reasonably high sensitivity and specificity in predicting the course of the illness. A line up of inflammatory, proteomic, gene expression and micro-RNA based biomarkers has been studied in association with KD. The commonly used inflammatory markers e.g. erythrocyte sedimentation rate (ESR), C-reactive protein (CRP), and total leucocyte counts (TLC) lack specificity for KD. Proteomic studies are based on the identification of specific proteins in serum, plasma and urine by gel electrophoresis. A host of genetic studies have identified genes associated with KD and some of these genes can predict the course and coronary outcomes in the affected individuals. Most of these tests are in the early stages of their development and some of these can predict the course, propensity to develop coronary artery sequelae, intravenous immunoglobulin (IVIg) resistance and the severity of the illness in a patient. Development of clinical criteria based on these tests will improve our diagnostic acumen and aid in early identification and prevention of cardiovascular complications.

## Introduction

Kawasaki disease (KD) is a common childhood vasculitis. The disease was first described in Japanese children in 1967 by a Japanese pediatrician, Dr. Tomisaku Kawasaki (1). The highest incidence is seen in Japan, Korea and Taiwan (2). However, the disease is now being reported world over including several developing countries like India (3–7).

The diagnosis of KD is essentially clinical (8). The clinical features mimic many self-resolving exanthematous febrile illnesses of childhood (e.g., measles, adenoviral infection, scarlet fever, dengue fever). These illnesses share some common clinical features with KD like fever, rash, mucocutaneous manifestations, lymphadenopathy, and elevated inflammatory parameters. The diagnosis of KD can be easily missed if the cascade of clinical findings goes unrecognized especially in cases of incomplete KD. Things can get even more complicated when KD occurs in association with an infection. The associated coronary artery abnormalities (CAAs) may go undetected and can have significant long-term implications if treatment is not initiated at the right time. Unlike other vasculitides and other rheumatological disorders, there are no pathognomonic laboratory tests for diagnosis of this condition. For the treating clinician, it is often difficult to confirm a diagnosis of KD on the bedside. It is, therefore, important to establish a set of laboratory markers that are sensitive, specific, reproducible and help the treating pediatrician in arriving at a diagnosis. As intravenous immunoglobulin (IVIg) is an expensive product, it is imperative that it be used only in situations where it is definitely indicated. However, in a setting of incomplete KD, the pediatrician often faces the dilemma of under-treatment with its attendant risks of CAAs vs. using IVIg in circumstances where it may not really be indicated.

Several biomarkers have been studied in association with KD and some of these have been shown to be predictive of resistance to IVIg while others may be indicative of an increased risk of development of CAAs. This manuscript overviews some of the important biomarkers that have been studied in KD and highlights their role in the diagnosis and assessment of disease severity.

## Inflammatory Biomarkers (Table 1)

These are the conventional biomarkers that mirror inflammatory activity and are not specific to KD. Erythrocyte sedimentation rate (ESR) is consistently elevated during the acute phase of KD but may be unreliable as a marker of disease activity after the administration of IVIG (9–11). There is neutrophilic leukocytosis during the acute phase of the disease and the degree of leukocytosis has been correlated with myocardial dysfunction (10, 12). Thrombocytosis, usually seen after the completion of the first week of illness, is a marker of ongoing inflammation. Persistent thrombocytosis has been linked to the development of CAAs but the association is tenuous (9, 18). C-reactive protein (CRP) is known to have a significant association with disease severity and the development of CAAs (9, 10, 12). Procalcitonin levels have been shown to be elevated during the acute phase and this rise is especially marked in children with resistance to IVIg (13, 16). Peripheral blood eosinophilia (PBE) (17) and low albumin has been associated with increased risk of IVIG resistance and coronary complications (14). Low mean platelet volume (MPV) (15), platelet distribution width (PDW) (19), and platelet-derived microparticles (PDMP) (20) have been shown to be markers of platelet activation and inflammation in acute stages of KD. However, their use as biomarkers for the disease requires replication of results across different populations.

**Table 1:** Inflammatory biomarkers of Kawasaki disease.

**Parameter**	**Normal values**	**Comment**	**References**
Erythrocyte sedimentation rate (ESR)	0–22 mm/h	•Increased in acute phases •Unreliable for monitoring response to IVIG therapy	(6–8)
Total leucocyte count(TLC)	4–11 × 109/L	•Higher counts associated with higher risk of CAAs •High in patients with delayed diagnoses of KD	(9–12)
Platelet count	150–400 × 109/L	Increased in acute stage and prolonged thrombocytosis associated with increased risk of CAAs	(6, 13)
Mean platelet volume(MPV)	7–11 fl	Low values increase the likelihood of CAAs	(14)
Platelet distribution width(PDW)	10.0–17.9%	High values suggest platelet activation and increase the likelihood of CAAs	(15)
C-Reactive protein(CRP)	<10 mg/L	Prediction of cardiac sequelae, age-dependent prognosis	(10, 11, 13)
Procalcitonin	<0.15 ng/mL	Increased in acute stage; will help differentiate acute KD from viral infections	(16, 17)
Peripheral blood eosinophilia (PBE)	0.0–6.0%	Higher rates in acute stages of incomplete KD; may be helpful in clinical setting of incomplete KD	(18)

### Summary

Inflammatory markers are largely nonspecific as these are also elevated in many other inflammatory and infective conditions. In a clinical setting of KD, while these biomarkers can reflect ongoing inflammation, they are of limited use in arriving at a definitive diagnosis.

## Immunological Markers (Table 2)

### Cellular Markers

#### Innate Immunity

Myeloid dendritic cells (mDCs), which serve as a bridge between adaptive and innate immunity, have been shown to be decreased in acute KD. Takahashi et al. (21) have suggested that decreased levels are due to either recirculation into affected tissues or increased peripheral destruction in cases of acute KD. Furukawa et al. (22) studied CD14+ macrophages in KD patients and showed that they were higher in those with CAAs, thereby suggesting that absolute counts of CD14+ monocytes can be a marker of the severity of KD. These studies point toward a possible dysfunction in the innate immunity axis which could contribute to the inflammatory upregulation seen in acute stages of KD.

**Table 2:** Immunological biomarkers of Kawasaki disease.

**Cellular markers**	**Biological functions**	**Comments**	**References**
CD8 T cells	Cytotoxic T cell	Decrease in acute KD; shown to sequester in inflamed coronary arteries, functionally suppressed	(21–23)
Th1 cells	Regulate cellular immunity by secreting IL-2 and IFN-γ	Downregulated in acute KD	(24)
Th2 cells	Regulate humoral immunity by secreting IL-4, IL-5, IL-6 and IL-10	Downregulated in acute KD and involved in response to IVIG	(21, 24)
CD14+ monocytes	Produce TNFα, IL-6, IL-1	Increased in acute stages and in association with CAAs	(21)
CD69+CD8T cells	Early activation marker for T cells	Increased in acute KD; marker to determine disease progression, treatment response, and convalescence in acute KD	(25)
Effector memory T-cells (Tem)	Found in the peripheral circulation and tissues; provide the immune system with “memory” against previously encountered pathogens.	Increase after IVIG treatment	(25)
Regulatory T cells (Treg)	Maintain tolerance to self-antigens	Decreased in acute KD, Increase after IVIG treatment	(25, 26)
Central memory T-cells (Tcm)	Found in the lymph nodes and in the peripheral circulation, provide the immune system with “memory” against previously encountered pathogens.	Increase in acute KD	(25)
Myeloid and plasmocytoid dendritic cells (DC	Most potent antigen presenting cells that initiates T-cell activation.	mDC increase in acute KDNo increase in pDC	(20)
Th17 proportions	Regulate inflammation by secreting IL-17	Decreased in acute KD, Increase after IVIG treatment	(27)
IFN-Y and IL-2	Th1 cytokine	Elevated in acute KD	(24)
IL-4, IL-10	Th2 cytokine	Elevated in acute KD	(24)
IL-6	Important mediator of the acute phase response	Upregulated in acute stages, more elevated in IVIG refractory cases	(24)
IL-17A/F, ROR-gt	Induce IL-6 production	Upregulated in acute stages; responsible for signs of inflammation	(28)
TGF-b	Marker of macrophage activation	Higher in acute stages, associated with CAAs	(29)
TNFa	Mediate endothelial cell activation	Increase in acute KD, role in CAAs	(30)
CXCL10 (IP-10)	Th1 associated chemokine	Upregulated in acute KD	(31, 32)
CCL-2	Th2 associated chemokine	Activation in acute KD	(31, 32)

#### Acquired Immunity

Studies have shown a decreased number of CD8 T cells during acute stages of KD (23). Immunohistochemistry studies in coronary arteries of KD patients at autopsy have shown that CD8 T cells preferentially sequester in the coronary arteries and are responsible for the inflammatory vasculitis (24, 26). Ehara et al. (27) showed that markers of early T cell activation [CD69(+)CD8T cells] increased in acute stages and can be used as a marker of disease progression and response to IVIg. Helper T cells (Th1 and Th2) have been shown to be upregulated during acute stages (22, 25). There is an apparent imbalance of T helper 17 cells (Th17) and regulatory T cells (Tregs) in acute KD. Th17 proportions have been shown to be upregulated while Treg proportions were found to be downregulated during the acute phase of KD (33).

#### Soluble Markers

Soluble markers of inflammation have been studied at great length in association with KD. Plasma levels of Th1 (IFN-Y, IL-12) and Th2 (IL-4, IL-13) cytokines have been shown to be elevated during acute stages of KD (34). Multiple pro-inflammatory and anti-inflammatory cytokines have been studied in acute stages of KD, but none of these has been standardized as a biomarker of KD (29). Zhou et al. (28) showed a close relation between levels of vascular endothelial growth factor (VEGF), IL-6, and development of CAAs. TGF-β signaling has been implicated in the development of coronary artery aneurysms (29). Th1-associated (CXCL10) and Th2-associated chemokines (CCL2) are elevated in acute stages of KD and have been shown to decrease with IVIG (35, 36). Tumor Necrosis Factor α (TNFα) has a role in the recruitment of inflammatory cells to coronary endothelium and has been shown to have a role in the development of CAAs. TNFα blocking agents have been extensively studied as a therapy for KD (37).

##### Summary

Data on immunological markers in KD are derived from studies on relatively small groups of patients. The significance of these biomarkers in predicting the disease course, response to therapy and complications is not clear and needs confirmation across a different population.

## Proteomic Biomarkers (Table 3)

Several proteins have been studied in KD.

**Table 3:** Proteomic biomarkers of Kawasaki disease.

**Protein**	**Biological function**	**Caveats**	**Limitations**	**References**
NT-pro BNP	Marker of myocardial damage; increased in response to cardiac dilatation and neuro-humoral factors	Higher values in CAAs and can predict IVIG resistance	Non-specific test • Can be elevated in other causes of diastolic dysfunction • Serum values vary with age	(38–44)
Suppression of tumorigenicity 2(sST2)	Member of the IL 1 receptor family and reflect cardiovascular stress and fibrosis	Elevated in acute stages of KD Correlate with impaired myocardial relaxation	Prognostic significance of sST2 levels in acute KD is unknown	(45)
Cardiac troponin I (cTnI)r	Marker of myocardial damage	Elevated in acute stages	Non-specific marker	(46, 47)
Periostin	Matricellular protein that plays a role in vascular and cardiac responses to injury	Upregulated 11-fold in acute and chronic KD coronary arteries	Non-specific	(48)
Gamma-glutamyl transferase(GGT) and Alanine transferase (ALT)	Biomarkers of cardiocyte inflammation	Increased in acute stages of KD	Non-specific	(45)
Clusterin	Component of high density lipoproteins; role in maintaining integrity of coronary endothelium	Values <12 mg/L associated with CAAs occurrence in KD patients	Need validation via larger studies	(49)
Thrombospondin (TSP-1 and TSP-2)	Involved in cardiovascular inflammation and maintaining the integrity and function of cardiac structures	• Elevated in acute KD • Associated with high risk of IVIg resistance	Need larger studies for validation	(50)
Fibrinogen beta and gamma chains	Cleavage products of fibrinogen and fibrin regulate systemic inflammation	Elevated in acute KD	Non-specific markers of inflammation	(49)
CD5 antigen-like precursor (CD5L)	Marker of acute inflammation	Increased in acute KD	Non-specific markers of inflammation	(49)
Nitric oxide synthases (iNOS)	NO has an important role in maintaining vascular tone and integrity of vessels	Correlate with the severity and progression of CAA	Non-specific marker of inflammation	(51)
Periostin	Matricellular protein of coronary endothelium	KD patients have significantly elevated serum values compared with febrile controls	Tissue based tests are difficult in clinical settings	(44)
Lipopolysaccharide-binding protein (LBP)	Markers of inflammation	Higher in acute KD	Need validation in larger studies	(52)
Leucine-rich alpha-2-glycoprotein (LRG1)	Markers of inflammation	Higher in acute KD	Need validation in larger studies	(52)
Angiotensinogen (AGT)	Markers of inflammation	Higher in acute KD	Need validation in larger studies	(52)
Tenacin- C	Extracellular matrix glycoprotein that is upregulated at sites of tissue injury and inflammation	Useful biomarker to predict the risk of developing CAAs and IVIg resistance	Need validation in larger studies	(53)
Urine protein markers: • Filamin • Talin • Complement regulator CSMD3 • Immune pattern recognition receptor muclin • Immune cytokine protease meprin A	Markers of inflammation	• Higher in acute KD • Non-invasive biomarkers of KD	Need validations via larger studies	(54)

### N-terminal Prohormone of Brain Natriuretic Peptide (NT-proBNP)

NT-proBNP has been used as a marker of myocardial damage in KD. It is a marker of cardiomyocyte stress imposed by pressure or volume overload and is increased in response to cardiac dilatation and neuro-humoral factors. According to Dahdah et al. (31), myocardial involvement in acute KD is universal from the histological and functional perspectives and hence the role of NT-proBNP as a potential biomarker has been extensively studied. The interpretation of serum NT-proBNP levels in children is difficult because these are age-dependent, being highest in infancy and decreasing thereafter. The sensitivity and specificity of cut-off values as a biomarker for KD are derived from receiver operating characteristics (ROC) analysis, the upper normal limit for age, and Z-scores for age. Shiraishi et al. (32) showed that the sensitivity and specificity of diagnosing acute KD were 97.8 and 47%, respectively, at a *z* score > 2. A meta-analysis on NT-proBNP in KD has substantiated its use as a diagnostic marker and cut-off values between 96 and 260 pg/ml have been shown to have sensitivity between 66 and 98% in identifying cases of KD (30). NT-proBNP levels are higher in patients with CAAs (values 515–1,300 pg/ml have a sensitivity and specificity of 73–95 and 61–85%, respectively) (38) and can predict IVIG resistance. A value between 629 and 1,300 pg/ml has a high sensitivity (70–79%) as well as specificity (58–77%) for diagnosis of KD (39, 40). From our center, we have reported a cut-off at 1,025 pg/mL for NT-proBNP levels which has a sensitivity of 88% and specificity of 96% (41) in the acute phase of KD.

### Other Cardiovascular Biomarkers

Suppression of tumorigenicity-2 (sST2) is a member of the interleukin 1 (IL-1) receptor family and reflects cardiovascular stress and fibrosis. It is elevated in acute stages of KD and the levels correlate with impaired myocardial relaxation. However, the prognostic significance in acute KD is unclear (42).

Kim et al. (43) have described cardiac troponin I (cTnI) in relation with KD and showed a significant increase in the level of cTnI in the acute stage of KD. Periostin is a matricellular protein that mediates responses associated with cardiovascular injury. Reindel et al. (44) have shown that periostin gets upregulated in coronary arteries during the acute and chronic phases of KD when compared to other febrile controls. Gamma-glutamyl transferase (GGT) and alanine transferase (ALT) have also been studied as biomarkers of cardiac inflammation. However, these are rather nonspecific and are not a reliable marker for KD *per se* (42).

### Thrombospondin (TSP-1 and TSP-2)

TSP-1 and TSP-2 are proteins involved in cardiovascular inflammation and maintaining the integrity and function of cardiac structures. These have been shown to be elevated in the acute phase of KD in comparison with other febrile controls and higher values are seen in those with IVIg resistance. With a cut-off value of 31.50 ng/mL, the sensitivity of TSP-2 has been shown to be 82.35% and specificity 64.81% in predicting IVIG resistance in acute KD (45).

### Other Proteins

Clusterin is a part of the high-density lipoproteins (HDL) and has a role in many physiologic processes including maintaining the integrity of coronary artery walls. Plasma clusterin levels have been studied in KD and values lower than 12 mg/L have been associated with the occurrence of CAAs in KD patients (46). Yu et al. (46, 55) have studied several proteins of fibrinogen cascade in KD. It was found that these were increased in patients with KD and may serve as a good biomarker of KD. The expression of nitric oxide synthases (iNOS) has been shown to correlate with the extent of coronary damage and progression of CAAs in patients with acute KD (47). Tenascin- C (TN-C) is a marker of tissue injury and inflammation and has a role in the maintenance of the extracellular matrix of cardiac tissue. Okuma et al. (48) have studied serum TN-C level in association with the risk of developing CAAs and resistance to IVIg therapy in the acute phase of KD.

#### Summary

Several protein biomarkers have been studied in relation to KD but most of these studies are based in small cohorts of patients at a single center. NT-proBNP is widely believed to be a useful marker for confirmation of the diagnosis of KD at the bedside. The levels of NT-proBNP in KD, however, may overlap those in other febrile illnesses with cardiac dysfunction. Other biomarkers are still in their early stages of discovery and will require validation in larger populations before the results can be used in clinical settings.

### Urine Biomarkers

Kentsis et al. (49) studied urine protein biomarkers in relation with KD and showed an abundance of markers of tissue injury (filamin and talin), complement regulator (CSMD3), cytokine protease (meprin A), and immune pattern recognition receptor (muclin) in the urine of affected patients. However, these results need replication in larger studies before these can be used as noninvasive biomarkers in KD.

## Genetic Studies in KD (Table 4)

A genetic basis of KD has been strongly considered taking into account the geographical differences in the incidence and high risk of occurrence in family members. Incidence of KD in Japan, Korea and Taiwan is more than 10 times the incidence in Western countries (50–53). This difference is a pointer toward a possible genetic susceptibility or may reflect environmental or lifestyle differences amongst these populations. A higher incidence is also known amongst Japanese Americans settled in Hawaii and this is comparable with the incidence in Japan, further pointing toward a possible genetic association of the disease (75). Studies have shown that the risk of developing KD in siblings of a KD patient is 10–30-fold higher as compared to the general population (76). Two types of approaches have been utilized for studying the genetic basis of KD:

**Table 4:** Genetic biomarkers of Kawasaki disease.

**Gene**	**Biological function**	**Study**	**Year and country**	**Ethnicity**	**Polymorphism**	**Conclusion**	**References**
ITPKC	Calcium channel modulator and regulates calcium release from ER, Acts as negative regulator of T cell activation	Wang et al.	2014, China	Asian	rs2720378, rs2069762	Higher risk of developing KD	(56)
		Peng et al.	2012, China	Asian	rs2290692	Higher risk of developing KD	(57)
		Kou et al.	2011, Taiwan	Asian	rs28493229	Higher risk of developing KD and higher risk of CAAs	(58)
ORAI1	Involved in calcium influx into T-cells and activation of the Ca2+/NFAT pathway, regulates immune system and inflammatory responses	Onouchi et al.	2016, Japan	Asian	rs3741596	Higher risk of developing KD	(59)
CD40	Activates immune system and is involved in immune and inflammatory responses	Lou et al.	2016, Japan	Asian	rs2736340, rs4813003, rs3818298	Higher risk of developing KD	(60)
		Cheng et al.	2015, China	Asian	rs1801274	Higher risk of developing KD	(61)
		Onouchi et al.	2012, Japan	Asian	rs1535045, rs4813003	Higher risk of developing KD	(62)
BLK	Involved in signal transduction and phosphorylation of ITAM residues of Iga and Igb, Responsible for B cell activation	Lou et al.	2015, China	Asian	rs2736340	Higher risk of developing KD	(60)
		Chang et al.	2013, Taiwan	Asian	rs2736340	Higher risk of developing KD	(63)
		Lee et al.	2012, Taiwan	Asian	rs2618476, rs2736340	Higher risk of developing KD	(64)
FCGR2A	Involved in metabolism and turnover of circulating IgG, Required for phagocytosis and clearing of immune complexes	Duan et al.	2014, China	Asian	rs1801274	Higher risk of developing KD	(65)
		Khor et al.	2013, Singapore	Mixed	rs1801274	Higher risk of developing KD	(66)
		Yan et al.	2013, China	Asian	rs1801274	Higher risk of developing CAAs in KD	(67)
CASP3	Involved in cell apoptosis, regulates cellular processes in T cells	Wang et al.	2014, China	Asian	rs2069762, rs2720378	Higher risk of developing KD	(68)
		Onouchi et al.	2010, Japan	Asian	rs72689236	Higher risk of developing KD	(69)
		Kou et al.	2011, Taiwan	Asian	rs72689236	Higher risk of developing KD	(70)
TGFβR2	Regulation of gene transcription	Choi et al.	2012, Korea	Asian		Higher risk of developing KD	(71)
SMAD3	Signal transducer and transcriptional modulator, involved in down-regulation of T-cells and cardiovascular remodeling	Kuo et al.	2011, Taiwan	Asian	rs1438386	Higher risk of developing KD, but not to CAAs	(72)
		Peng et al.	2016, China	Asian	rs1438386	Higher risk of developing KD	(73)
ADAM17	Required for activation of notch signaling pathway and processing	Peng et al.	2016, China	Asian	rs6705408	Higher risk of developing KD and development of CAAs	(73)
MMP-11	Causes breakdown of extracellular matrix	Ban et al.	2010 Korea	Asian	rs738792	Higher risk of developing KD	(74)

1. Candidate gene approach
2. Genome-wide approach

### Candidate Gene Approach

Candidate gene studies are the well-known approach to study genes associated with KD based on their function in the pathophysiology of the disease.

#### HLA Genes

Early genetic studies on KD were focused on HLA alleles. HLA class 1 antigens have been studied in great detail in various populations (54, 77). Significant predominant association of HLA-Bw54 has been found in Japanese KD population while single nucleotide polymorphisms (SNP) located in the *HLA-E* gene was suggested to be associated with KD in the Han Chinese population (77). A positive association was found between HLA-Bw51 and KD in the White and Jewish population while HLA-Bw51 was reported to be negatively associated in the Korean population. In a recent GWAS, association with HLA class II antigens peaked at the intergenic region between HLA-DQB2 and HLA-DOB (78). SNPs in HLA class III region have also been described in association with KD (79). TNF∞ expression has been shown to be elevated in association with CAAs in KD in Korean, Taiwanese, Chinese and Caucasian populations, but these results could not be replicated in studies from Japan (80).

#### Non-HLA Genes

Burns et al. (78) found an SNP in the promoter region of the IL-4 gene to be asymmetrically transmitted in children with KD. MHC-class-I-chain-related gene A (MICA) alleles were reported as biomarkers for evaluation of coronary aneurysms in KD. Lower frequency of MICA allele 276 A4 was reported in KD patients with aneurysms (81).

### Genome-Wide Approach

#### Genome-Wide Linkage Analyses (GWLS)

GWLS were used to map the genetic loci of diseases by analyses of related individuals through the logarithm of odds (LOD) score. The first GWLS on KD was done by Onouchi et al. (82) who studied 79 families of children with KD and analyzed 75 sibling pairs. They identified 10 chromosomal loci with positive linkage, among which the 12q24 region showed the most significant evidence of linkage. GWLS studies identified Inositol 1,4,5-trisphosphate 3-kinase C (ITPKC) gene in association with risk of KD (83).

#### Genome-Wide Association Studies (GWAS)

GWAS are based on the analysis of a genome-wide set of genetic variants typically SNP in affected individuals and healthy controls to see if a variant is repeatedly associated with a disease. GWAS studies on KD have identified several genes in association with the disease. The significant susceptibility genes identified include caspase-3 (CASP3) (69), calcium release-activated calcium modulator 1 (ORAI1) (59), ATP-binding cassette sub-family C member 4 gene(ABCC4) (84), Fc Fragment of IgG Receptor IIa (FCGR2A) (66), Transforming growth factor β pathway (TGFB2, TGFBR2, and SMAD3) (85), B lymphocyte kinase (BLK) (63), matrix metalloproteinase 11 (MMP-11) (74), and CD40 (86).

### Genes Associated With B-Cell Signaling

#### CD40

CD40L–CD40 interaction is known in relation to triggering and progression of acute coronary syndromes (87). Higher CD40 ligand expression on CD4 T-cells of KD patients as compared to febrile controls has been reported by a previous study. This over-expression decreased after IVIg administration (88). SNPs around CD40 showed association with KD susceptibility in Japanese (rs4813003, located 4.9 Kb downstream) and Taiwanese (rs1569723, located 4.8 Kb upstream) population. GWAS from Taiwan and Japan have also shown the association of SNPs of *BLK* and *CD40* in the pathogenesis of KD (66).

#### B Lymphoid Tyrosine Kinase (BLK)

BLK has a role in signal transduction of B cells (64). GWAS from Taiwan and Japan have shown the association of SNPs of *BLK* and *CD40* in the pathogenesis of KD (68). Kawasaki Disease Genetics Consortium (2013) confirmed the association of BLK with KD susceptibility in Korean and European population (89).

#### Fc Fragment of IgG Receptor IIa (FCGR2A)

*FCGR2A* is present on Fc region of IgG and encodes for cell surface receptor protein on phagocytic cells. GWAS done in five independent centers have confirmed the association of *FCGR2A* with KD susceptibility (66). Associations of SNPs rs2736340, rs4813003, rs3818298, and rs1801274 with KD were reported in a Han Chinese population (60, 66).

### T-Cell Activation Genes

#### Inositol 1,4,5-Trisphosphate 3-kinase (ITPK) Enzyme Gene

*ITPKC* is involved in Ca^2+^/NFAT pathway and acts as a potential candidate for immune activation and T-cell receptor signaling (68, 83). Association of functional SNP (rs28493229) in ITPKC with development of KD and CAA risk was first reported in Japanese and American children (83). However, no significant association was found between rs28493229 and KD risk in Taiwanese patients (90). Significant associations of C-allele of rs28493229 with KD and BCG scar reactivation in the acute stage were reported by Lin et al. (56).

#### Calcium Release-Activated Calcium Channel Protein 1 (ORAI1)

*ORAI1* is a Ca^2+^ channel protein involved in T cell regulation and inflammatory responses. Onouchi et al. (59) showed a significant non-synonymous association of SNP (rs3741596) in exon 2 of the ORAI1 gene with a high risk of KD in Japan. Another study reported a rare variant (rs141919534) in association with KD (91).

### Genes Associated With the Apoptotic Pathway

#### Caspase 3 (CASP3)

*CASP3* gene is an execution-phase caspase involved in apoptosis of immune cells. Various SNPs including rs2720378, rs72689236, and rs113420705 have been reported in association with KD (69). Kuo et al. found that SNP rs28493229 (*ITPKC*) and rs113420705 (*CASP3*) showed an increased risk of IVIG resistance and development of CAAs in Japanese but not in Taiwanese population (92).

#### TGF-β Signaling Pathway

The TGF-β pathway is an essential component of inflammation, T-cell activation and tissue remodeling. Genetic variations in *TGF-*β*2, TGF-*β*R2*, and *SMAD3* are associated with CAA formation and was confirmed in replication studies (85). SNPrs6550004 in TGF-βR2 and rs1495592 in SMAD5 showed significant associations with KD in the Korean population (93).

### Gene Expression Studies

MicroRNAs (miRNAs) are 20–26 nucleotides long non-coding single-stranded RNA segments that modify post-transcriptional mRNA gene expression. Shimizu et al. (94) showed 6 miRNAs (miR-143, miR-199b-5p, miR-618, miR-223, miR-145, and miR-145) that were differentially expressed in acute KD. In another study, miRNA-200c and miRNA-371-5p have also been studied as diagnostic biomarkers of KD (54). miR-27b suppresses endothelial cell proliferation and has been studied as a therapeutic target in patients of acute KD (95).

- In a recent multicenter study by Wright et al. (96), the use of whole-blood gene expression patterns was explored and a 13-transcript blood gene expression signature has been developed to distinguish KD from other infectious and inflammatory febrile illnesses in the first week of illness.
- Whole genome sequencing in a family with KD has also shown genetic variations in the toll-like receptor-6 (TLR6) gene in their two affected children (97).

**Summary:** Most of the genetic studies have been carried out in small cohorts of patients with KD and the results are not reproducible across different populations and ethnicities. These studies require validations in larger and multinational cohorts with additional case-control sets for a better understanding of the genetic profiles.

## Conclusions

The need for a robust set of biochemical biomarkers to validate the diagnosis of KD in the clinical setting has become the need of the hour. Clinical application of these biomarkers is limited. Inflammatory parameters can, at best, facilitate confirmation of a clinical diagnosis of KD but none of these is pathognomonic of KD. Further, these markers have very low specificity for the diagnosis of KD. The newer proteomic studies have identified some biomarkers in association with KD but these also need validation across different populations before these can be used for confirming a diagnosis of KD. Genome-wide studies, linkage studies and miRNA-based biomarkers are still in their early stage of development and fall short of being a diagnostic test for this enigmatic condition. These genetic markers are pointers toward a diagnostic panel for KD but clearly, these are early days and much more work needs to be done before a robust laboratory diagnostic test can be established.

## Author Contributions

HC, JN, RK: Literature review, interpretation of data and draft of the manuscript. VP: critical review and editing of the manuscript. DS: critical review of studies cited in the manuscript, editing of manuscript. AR: concept and design of the manuscript, critical review of studies cited in the manuscript, editing of manuscript. SS: concept and design of the manuscript, critical review, and editing of the manuscript.

### Conflict of Interest Statement

The authors declare that the research was conducted in the absence of any commercial or financial relationships that could be construed as a potential conflict of interest.
